# Visual Thinking Strategies in medical education: a systematic review

**DOI:** 10.1186/s12909-023-04470-3

**Published:** 2023-07-27

**Authors:** Ana Rita Cerqueira, Ana Sofia Alves, Matilde Monteiro-Soares, Dabney Hailey, Domingos Loureiro, Sofia Baptista

**Affiliations:** 1 Faculty of Medicine, University of Porto, Alameda Prof. Hernâni Monteiro, 4200 – 319 Porto, Portugal; 2 Lordelo Do Ouro Family Health Unit, Agrupamento de Centros de Saúde Porto Ocidental, Rua de Serralves 20, 4150-701 Porto, Portugal; 3 Department of Community Medicine, Information and Health Decision Sciences (MEDCIDS), Faculty of Medicine, University of Porto, Alameda Prof. Hernâni Monteiro, 4200 – 319 Porto, Portugal; 4 RISE@ CINTESIS, Faculty of Medicine, University of Porto, Alameda Prof. Hernâni Monteiro, 4200 – 319 Porto, Portugal; 5 Portuguese Red Cross School of Health – Lisbon, Avenida de Ceuta, 1 Edifício Urbiceuta, 1300-125 Lisbon, Portugal; 6 Harvard Medical School and the Hailey Group, Boston, MA USA; 7 Faculty of Fine Arts, University of Porto, Av. de Rodrigues de Freitas 265, 4049–021 Porto, Portugal; 8 Agrupamento de Centros de Saúde Porto Ocidental, Rua Do Molhe 181, 4150-502 Porto, Portugal

**Keywords:** Visual thinking strategies, Undergraduate medical education, Postgraduate medical education, Art

## Abstract

**Background:**

Arts-based pedagogical tools have been increasingly incorporated into medical education. Visual Thinking Strategies (VTS) is a research-based, constructivist teaching methodology that aims to improve visual literacy, critical thinking, and communication skills through the process of investigating works of art. Harvard Medical School pioneered the application of VTS within medical education in 2004. While there are several studies investigating the use of VTS, there is a need to systematically assess the different programs that exist for medical education and their efficacy in improving relevant clinical skills. This systematic review aims to critically analyse the available evidence of the effectiveness of VTS in medical education to guide future research and provide a framework to adapt medical curricula.

**Methods:**

A systematic search of PubMed, PsycINFO, and Cochrane CENTRAL databases (through November 2022) was conducted to identify studies of VTS-based interventions in undergraduate and postgraduate medical education. Two reviewers independently screened citations for inclusion criteria, extracted data, and assessed risk of bias. The extracted data was then narratively synthesized.

**Results:**

Of 5759 unique citations, 10 studies met the inclusion criteria. After reference review, one additional study was included. Therefore, 11 studies were included in our review. Of these, eight reported VTS-based interventions for undergraduate medical students and three reported interventions in residency training, specifically in dermatology and ophthalmology. The main goal of most studies was to increase observational or visual diagnostic skills. Three of the studies in undergraduate medical education and two in postgraduate achieved a statistically significant improvement in observational skills in post-course evaluations. Some studies reported increased tolerance for ambiguity and empathy.

**Conclusions:**

Although the studies varied considerably in study design, learning objectives, and outcomes, findings consistently indicate that the VTS approach can serve as a vehicle to develop crucial clinical competencies, encouraging more in-depth visual analysis that could be applied when observing a patient. Despite some limitations of the included studies (lack of control groups, self-selection bias, or non-standard outcome measures), the results of this review provide support for greater inclusion of VTS training in the medical curriculum.

**Supplementary Information:**

The online version contains supplementary material available at 10.1186/s12909-023-04470-3.

## Background

Arts-based pedagogical tools have been increasingly incorporated into medical education. According to a recent and updated report, there are more than 125 programs arising from partnerships between art museums and medical schools [1]. Learning through art promotes the acquisition of a variety of important skills and competencies for clinical practice, such as observation skills, team building and communication skills, and cultural sensitivity [2, 3]. Furthermore, exposure of medical students to the humanities correlates with reduced burnout and with positive personal qualities, including empathy, tolerance for ambiguity, and emotional appraisal [4, 5]. Although there is evidence that some of these skills, specifically empathy, among healthcare practitioners correlate with better clinical outcomes [6], some studies suggest that the degree of empathy shown by medical students declines as they progress through their education [7]. These findings highlight the importance of incorporating intentional training in these skills into the medical curriculum.

Abigail Housen, a cognitive psychologist, and Philip Yenawine, an art museum educator, developed a research-based, constructivist art-based teaching methodology called Visual Thinking Strategies (VTS). VTS is the use of art to teach visual literacy, thinking, and communication skills [8]. VTS was not originally developed for medical education, but rather for museum education. During a VTS discussion, a facilitator uses a very specific facilitation protocol to steward participants as they explore a work of art together, sharing observations and interpretations. VTS was created over a ten-year iterative process of testing and data-driven revisions that began in 1991. The resulting protocol instructs facilitators to present a carefully selected image, allow a few moments to look at it silently before beginning the discussion, and then pose three specific research-tested questions: 1) “What is going on in this picture?”, 2)” What do you see that makes you say that?”, and 3) “What more can you find?”. Rigorous facilitation also includes listening carefully to what students say while maintaining a neutral stance, pointing to observations as students make comments, paraphrasing each comment, and linking related comments to surface commonalities and differences in interpretations [9, 10].

VTS has been successfully used in K-12 education programs [11, 12] and is currently being used in a wide variety of different settings, including in arts-based courses at medical schools and in continuing medical education [13–15]. Harvard Medical School’s ongoing course, “Training the Eye: Improving the Art of Physical Diagnosis”, pioneered the application of VTS within medical education in 2004 [15].

While there are several studies investigating the use of VTS, there is a need to systematically assess the different existing programs for medical education based on this learning methodology and their efficacy in improving relevant clinical skills. To our knowledge, this is the first systematic review of VTS in medical education. Our main goal is to critically analyse the available evidence of the effectiveness of VTS in medical education to guide future research and provide a framework for future directions in medical curricula. The research question that guided this review was: “Do VTS-based interventions for undergraduate and postgraduate medical students improve clinical skills?”.

## Methods

We conducted a systematic review according to the Preferred Reporting Items for Systematic Reviews and Meta-Analyses (PRISMA) recommendations [16]. Our protocol is registered on the International Prospective Register of Systematic Reviews (PROSPERO) (CRD42022366934).

### Data sources and searches

We searched PubMed, PsycINFO, and Cochrane CENTRAL (Cochrane Central Register of Controlled Trials) databases in November 2022.

Whenever possible, the search strategies (Additional file 1) used a combination of free text and database-specific subject headings to represent the concepts of medical education and Visual Thinking Strategies. There was a deliberate attempt to perform a high-sensitivity search in order to maximize yield.

Reference lists of studies meeting the inclusion criteria and studies included in retrieved reviews from our database search were manually reviewed for additional articles.

### Study selection

We imported citations from all databases into Rayyan QCRI [17]. Rayyan offers duplicate resolution for statistically likely duplicates by comparing the title, author, journal, and year. We used Rayyan to detect duplicates and then one author (ARC) manually resolved duplicates.

We included a study if it fulfilled the following criteria: *(i)* Population: medical students, interns, or residents; *(ii)* Intervention: VTS-based interventions (defined as any intervention that explicitly used this teaching method). No exclusion was made based on the outcome. Studies were not excluded based on their type of study design, but a study was excluded if it only described the VTS methodology without an effect assessment. Studies that had multiple arts-based interventions rather than only VTS were excluded. Articles prior to 1991 were also excluded, since the VTS methodology was not available before then.

Two reviewers (ARC and ASA) independently performed a title and abstract screen of all retrieved articles after employing the search strategy. Disagreements at this screening stage were resolved by consensus and studies included after screening were retrieved for full-text analysis. ARC and ASA independently performed a full-text review using inclusion/exclusion criteria. Any disagreements at this stage were similarly resolved by consensus.

### Data extraction, quality assessment and data synthesis

A data extraction form was created to extract predefined data points from each included study. Two reviewers (ARC and ASA) independently extracted qualitative and quantitative data from each reference, including study design, number and type of participants, number of sessions, duration, goal and format of VTS-based sessions, measurement, results, control group and assessment of overall study quality.

Study quality was assessed using the Joanna Briggs Institute (JBI) Critical Appraisal Checklists [18]. We used the JBI Checklist for Qualitative Research, JBI Checklist for Quasi-Experimental Studies, and JBI Checklist for Randomized Controlled Trials (RCT) to rate studies across various domains requiring a yes, no, unclear, or not applicable response, whereby items scored as ‘no’ were awarded zero points and items scored as ‘yes’ were awarded one point. We also used the Medical Education Research Study Quality Instrument (MERSQI) [19], a ten-item instrument designed to assess the methodological quality of experimental, quasi-experimental, and observational medical education research studies. The ten items reflect six domains of study quality: study design, sampling, data type (subjective or objective), validity of assessments, data analysis, and outcomes [19]. Possible total MERSQI scores range from 5 to 18. MERSQI score is a previously validated tool with strong content, criterion, and predictive validity evidence to assess the quality of quantitative studies [19–21]. Differences in classification were resolved by consensus between the reviewers.

The extracted data was narratively synthesized reporting on the characteristics of VTS-based interventions and key findings. The results were structured dividing studies reporting VTS-based interventions in undergraduate medical education and studies reporting VTS-based interventions in residency.

## Results

### Search results

**Fig. 1:**
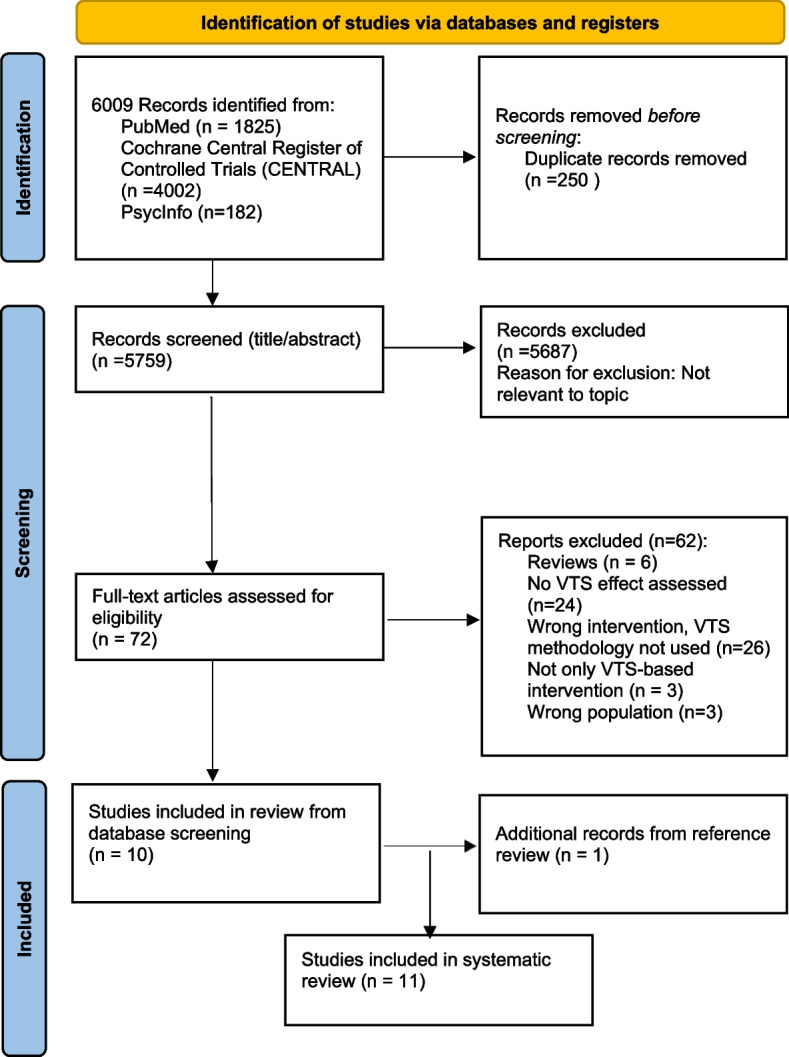
PRISMA flow diagram

The search process is depicted in the PRISMA flow diagram (Fig. 1). A total of 6009 citations were identified initially by our electronic database search strategy; 250 duplicates were removed retrieving a total of 5759 unique citations for review. Of these, 72 full-text articles were reviewed, and 10 studies met our eligibility criteria. After reviewing references of these articles and studies included in six reviews excluded at the full-text review stage, we included one additional study. We therefore included a total of 11 studies in the final qualitative synthesis.

### Study characteristics

Study characteristics are presented in Table 1. The included studies were published between 2008 and 2022. Seven studies (64%) were based in the United States of America (USA) [22–28], two in Canada [29, 30], one in Israel [31], and one in the United Kingdom [32]. Seventy-three percent (*n* = 8) of studies reported VTS-based interventions for undergraduate medical students [22–24, 26, 28–31] and 27% (*n* = 3) reported interventions in residency training, specifically in dermatology training (*n* = 2) [25, 32] and in ophthalmology training (*n* = 1) [27]. Fifty-five percent (*n* = 6) of the studies utilized pretest–posttest designs [23–27, 32]. Three studies that were included were primarily qualitative in nature [28–30], while nine incorporated quantitative methods [22–27, 29, 31, 32]. Two of the studies, both with undergraduate medical students, utilized control groups [22, 26].

**Table 1:** Study characteristics – Visual Thinking Strategies in Medical Education

**Study**	**Study design**	**Number of participants**	**Participants**	**No. of sessions, duration**	**Goal**	**Format**	**Measurement**	**Results**	**Control group**	**MERSQI score**
Naghshineh et al., 2008 [22]	Prospective, partially randomized study design with pre- vs. post-course evaluations	58 Intervention (*n* = 24) Control (*n* = 34)	1st-2nd year Medical and Dental students	9 weekly, 2.5-h sessions	Improve Visual Diagnostic Skills	Elective pre-clinical course: “Training the Eye: Improving the Art of Physical Diagnosis” at the Boston Museum of Fine Arts (MFA), facilitated by art educators trained in VTS, followed by a lecture linking visual arts concepts with physical diagnosis	Comparison of post-course vs. pre-course mean frequency of accurate observations	Increased observation skills. Increased use of fine arts concepts linked to physical findings in descriptions of clinical images. Improvement in participants who attended 8 or more sessions compared to 7 or fewer	Yes	12.5
Klugman et al., 2011[23]	Quasi-experimental	32 (18 medical students)	1st-3rd year Medical Students and Nursing Students	3 weekly, 90-min sessions	Improve physical observation skills, increase tolerance for ambiguity, and increase interest in learning communication skills	Sessions at an art museum led by museum educators trained in VTS as a part of an enhancement program	Comparison of pre and posttest evaluations	Increased total time spent looking at art and patient images. Increased number of words used to describe and number of observations made. Increased tolerance for ambiguity and positive views toward healthcare professional communication skills. No significant differences between medical and nursing students	No	11
Jasani & Saks, 2013 [24]	Quasi-experimental	110	3rd-year Medical Students	1 session, 3 h	Improve observation skills in clinical diagnosis	Classroom discussion about fine art images, facilitated by a 4th-year medical student with interest in the visual arts. Part of a required course	Comparison of pre and posttest evaluations of patients’ photographs descriptions	The mean number of observations between pre- and posttests was not significantly different. Decreased use of subjective terminology. Increased scope of interpretations, use of speculative thinking, and visual analogies on descriptions. Increased mindfulness and clinical observation skills on student feedback	No	10
Huang et al., 2016 [25]	Quasi-experimental	27	PGY- 2–5 Dermatology residents	4 sessions, 7,5 h total, over a 2-month period	Improve observation skills	Mandatory course at the Museum of Fine Arts Boston, led by 2 professional arts educators and 2 dermatology faculty, all with formal VTS training	Comparison of pre- and posttest descriptions of clinical and art images	The overall number of observations made by residents on image tests significantly increased	No	11
Bentwich & Gilbey, 2017 [31]	Quasi-experimental	67	1st-year Medical Students	1 session, 90 min	Increase tolerance of ambiguity and increase empathy	Classroom combined lecture and interactive discussion about art images, led by a physician with an interest in art. Performed within a mandatory course	Subjective participant feedback (post-intervention survey)	Increased acceptance of multiple meanings (tolerance of ambiguity) and improved visual observation skills. Impact on the ability to feel the suffering of others and on teamwork. High correlation between increased acceptance of multiple meanings and increased empathy	No	7.5
Allison et al., 2017 [30]	Qualitative	8	1st and 2nd-year Medical Students	1 session, 20 min	Improve medical students’ understanding of the social determinants of health	Analysis of a street art mural in Nepal depicting the lives of ordinary Nepalis, facilitated by 3 faculty members.This session was a part of a pre-clerkship elective for medical students from Canada	Transcripts from sessions	Consolidation of complex community health concepts. Deeper understanding of the social determinants of health in Nepal	No	N/A (qualitative study)
Ho Tiu et al., 2019 [32]	Quasi-experimental	10	PGY-1/PGY-2 dermatology trainees	7 sessions	Improve clinical skills	Visual literacy training course taking part in art galleries and facilitated by an art historian	Participant satisfaction surveys and pre- and post-course assessments	Improved clinical observational skills. Expanded vocabulary and descriptive ability for clinical material. Increased clinical confidence and professional development across managing ambiguity, communication, respect, and reflective practice. Most pronounced impact on first-year trainees and on females	No	11
Visscher et al., 2019 [29]	Qualitative	50	3rd-year Medical Students	1 session, 45 min	Improve medical students’ understanding of the radiology profession	Artworks depicting radiology encounters with patients, presented as digital images, were analysed, moderated by a VTS facilitator. This session was a part of a one-week radiology elective	Subjective participant feedback (post-session questionnaire) and transcribed audio recordings of sessions	Better understanding of the radiologists’ clinical roles. Reduced negative stereotypes of the radiology profession and of radiologists	No	N/A (qualitative study)
Agarwal et al., 2020 [26]	Quasi-experimental	101 Intervention *n* = 41 Control *n* = 60	1st-year Medical Students	Two 3-h sessions, over two weeks	Improve observation skills	Workshop at a university art museum, facilitated by museum educators. Participation was voluntary	Comparison of pre- and post-tests descriptions	Increased number of clinical and general patient observations and no statistically significant difference in the number of diagnostic comments or “self-deprecating remarks” Increased number of words used to describe clinical images Increased total time spent analysing and describing clinical images	Yes	11.5
Cole et al., 2020^a^ [27]	Single-arm trial	4	PGY-1/ PGY-2 ophthalmology residents	3, 60-min sessions	Increase observation skills and increase ability to apply these skills in clinical settings. Increase tolerance of ambiguity	Classroom observation and discussion of art images, facilitated by an art historian	Subjective participant feedback	Improved clinical and observational skills. Improved detection of visual elements, attention to descriptive detail, awareness of assumptions, and acceptance of multiple possible meanings	No	Unclear
Srivastava et al., 2022 [28]	Qualitative	29 students	Medical students	10 weekly,2.5 h sessions	Improve observation skills	A virtual “Training the Eye: improving the art of physical diagnosis” elective course. One session in person followed by 9 remote sessions through Zoom, using high-resolution images of artwork selected from museums around the world. Facilitated by a VTS trained art educator, Teaching Assistants, and the students themselves, who learned VTS facilitation during the course	Subjective participant feedback (post-course questionnaire)	Appreciation for deep looking and for the mindfulness aspects of the humanities curriculum. Better understanding of visual biases. 75% of students agreed or strongly agreed that the course objectives were able to be met virtually	No	N/A (qualitative study)

*N/A* Not applicable

^a^Only available as a conference abstract

#### Quality appraisal

Assessments of the risk of bias for each study are summarized in Additional file 2. The average quality score of the JBI critical appraisal checklist was 7/10 for qualitative studies, 6/9 for quasi-experimental studies, and the quality score for RCT was 8/13 with risk of bias due to lack of true randomization, lack of allocation concealment, absence of blinding, or incomplete follow-up.

The MERSQI score for each quantitative study is reported in Table 1. The maximum MERSQI score allowed is 18 points. The mean score was 11 points, the highest score for an article was 12.5, and the lowest was 7.5.

### Visual thinking strategies in undergraduate medical education

A variety of VTS-based interventions have been integrated into medical school education. The participants in the studies that were included were first to third-year medical students. The duration of the projects ranged from 1 to 10 sessions and from 20 min to 10 weeks. Seventy-five percent (*n* = 6) of the interventions were performed as a part of an elective course [22, 23, 26, 28–30], while two studies [24, 31] reported VTS-based sessions within a mandatory course.

Three studies [24, 29, 31] described VTS applied in a classroom setting and three studies [22, 23, 26] described interventions in art museums. Allison et al. described the use of VTS methods in a totally different setting: in the street. The students were instructed to analyse a street art mural in Nepal [30]. Srivastava et al. described a “Training the Eye: Improving the Art of Physical Diagnosis” course adaptation to online learning due to the COVID-19 pandemic [28]. Their study reported VTS exercises using high-resolution images of artwork from museums around the world through Zoom screen sharing.

The VTS-led exercises were facilitated by art or museum educators in three studies [22, 23, 26], while physicians or faculty members were facilitators in four studies [28–31]. One study [24] described a classroom discussion about eight fine art images, facilitated by a fourth-year medical student with an interest in the visual arts. Fifty percent of the studies (*n* = 4) [22, 23, 26, 28] reported that facilitators had specific training in VTS.

The main goal of the VTS-based interventions was to increase observational or visual diagnostic skills in five studies (63%) [22–24, 26, 28]. Four of these studies compared pre and post-course descriptions of different clinical images [24, 26] or clinical and art images [22, 23] to measure outcomes, while one [28] based its conclusions only on subjective feedback from students through a post-course questionnaire. Three of these five achieved a statistically significant increase in observational skills [22, 23, 26]. One study [24] reported no statistical improvement in observational skills between pre- and post-tests. One study [22] found a dose-dependent response between the number of art sessions participants attended and the level of improvement in observational skills, reporting that those who attended eight or more sessions (20 h) achieved a significantly greater increase in accurate observations compared to those who attended seven or fewer sessions.

Bentwich and Gilbey investigated other effects of VTS, specifically increased empathy, tolerance of ambiguity and teamwork ability [31]. In the post-intervention survey, participants reported an increase in multiple domains including acceptance of multiple possible meanings and visual observation ability. Only 16% of the students expressed support for the idea that the class contributed to their teamwork ability. Statistically significant moderate-to-high correlations were found between the contribution to ambiguity tolerance and contribution to empathy. Tolerance for ambiguity was addressed in another study [23], using a standardized test (Geller and colleagues’ variation of Budner’s Tolerance of Ambiguity Scale) [33], and a statistically significant increase in that endpoint was reported.

Visscher et al. utilized VTS to explore how representational paintings of radiology encounters with patients may influence medical students’ understanding and impression of radiologists and the radiology specialty. After the session, participants reported a better understanding of the clinical roles of radiologists and reduced negative stereotypes of radiology and radiologists [29]. Allison et al. investigated how VTS methods can be applied to explore the social determinants of health through the analysis of street art. The VTS-based exercise allowed participants to articulate their understanding of the social determinants of health in Nepal, suggesting that carefully looking at and reflecting on visual art can assist medical students to apply, analyse, and evaluate complex concepts in global health [30].

### Visual thinking strategies in residency

Three studies evaluated the efficacy of VTS specifically in residency [25, 27, 32]. The participants were dermatology [25, 32] or ophthalmology [27] residents. The study describing VTS-based intervention for ophthalmology residents was only available as a conference abstract [27]. None of the studies used a control group. One intervention was performed within a mandatory course [25], while there was no information regarding this question in the two remaining studies. The duration of the projects ranged from three to seven sessions. Improvement of observational skills was the main goal of all the interventions. Both studies reporting interventions for dermatology trainees were led at art museums, while the intervention for ophthalmology residents consisted of classroom observation and discussion of art images. Huang et al. described a course for 27 dermatology trainees led by two professional arts educators and two dermatology faculty members, all with formal VTS training [25]. The two other studies reported VTS-based exercises led by an art historian not mentioning formal VTS training [27, 32].

Dermatology residents achieved a significant increase in observational skills in both studies after the intervention [25, 32]. However, one study [25] reported a trend towards greater improvement of scores among participants of a higher PGY-level, while the other [32] reported that the course had the most pronounced impact on first-year trainees.

In the study for ophthalmology residents [27], only one of the four participants completed posttesting, so a pre-post analysis was not pursued, despite 75% (n = 3) of participants reporting that the training improved their clinical practice.

## Discussion

### Summary of evidence

This systematic review aimed at identifying how the VTS methodology is being incorporated into medical education and how effective it is. A qualitative synthesis of 11 eligible studies identified various VTS-based interventions being employed as pedagogical tools within undergraduate and postgraduate medical education. Although the studies had mixed outcomes for the use of VTS in medical education, most findings highlight that VTS improved observational skills, empathy, and tolerance to ambiguity.

In most studies, the main educational goal was to increase observational skills. Most studies did not score very high on our quality assessment. There was no truly randomized controlled trial, only two studies had a control group and most were limited in sample size. Therefore, it is not possible to exclude that there were confounding factors impacting the results and that some of the improvements could be explained solely by random factors or chance. All the studies were single-institution studies, impacting the external validity of our findings, and none assessed observational skills in a clinical setting. It is also important to note the heterogeneity in the measurement of the outcomes across the different studies.

Since many studies reported interventions within elective courses, there may have been a potential selection bias in the participant group. Students were evaluated immediately after the course, lacking long-term follow-up data to assess potential sustained effect of VTS in medical education. Finally, clinical outcomes were not measured, so there is no direct evidence that the skills that were evaluated can be transferred into improved patient care.

A significant number of cognitive biases have been described that can adversely influence clinical reasoning [34, 35], making VTS a good teaching tool as VTS open-ended questions form a basis for unbiased observation. Students are confronted with the unknown when they are encouraged to deeply analyse unfamiliar artworks, and they end up searching and describing more observed details [36].

It is important to note that the efficacy of this teaching method was demonstrated even in dermatology trainees, who may have increased visual aptitude from baseline and are receiving training that is heavily focused on the acquisition of visual skills [25].

Of note, the study that specifically focused on assessing the impact of VTS on tolerance for ambiguity and empathy [31] had no control group, results were based on a post-intervention survey and that study had the lowest MERSQI score (7.5).

By adapting VTS to different contexts and using carefully selected pieces of art, studies have suggested that VTS can also be effective in achieving outcomes other than those usually described, such as a better understanding of global health concepts [30] or a better understanding of the clinical roles of radiologists [29].

Most of the programs were offered as an elective course, and some were integrated within the existing curriculum. One study [24] highlighted that positive outcomes were achieved with VTS exercises implemented without a museum partnership or trained staff. Although a formal training in VTS is desirable, since the methodology is highly dependent on the experience and training of the facilitators, this may suggest that VTS have the potential for a more broaden appliance in medical education. There appears to be an impact deriving from even short exposure to VTS-based exercises; even those with a duration of 20 min for instance, and such evidence provides additional support for greater inclusion of VTS training in the medical curriculum. However, the dosage as to the number and frequency of VTS training sessions remains unclear.

As a result of the pandemic, telehealth has become a crucial element of medical practice and the virtual VTS course enabled parallels to be drawn between the virtual art observation exercises and the paradigm of telemedicine. Since students reported that there are some advantages stemming from using the Zoom platform, such as being able to analyse art from around the world and increased accessibility [28], a hybrid model could be considered in when designing future courses, in order to prepare students for virtual observation.

In their course and program evaluations, students noted skills stimulated by VTS techniques that are crucial in providing dedicated, patient-centred care and working as part of a team, including the appreciation of multiple perspectives, managing ambiguity, and learning not to jump to conclusions.

The extended discussion allows more time to reconsider early, fast assessments, and the paraphrasing and evidence-seeking help mitigate assumptions and biases they may initially make. This meticulous and contemplative process may well be applied to clinical practice, allowing physicians to focus on the patient as a whole and to observe, ask, and consider more before landing on a diagnosis.

Our results are similar to those from a recent systematic review examining visual art-based training in undergraduate medical education [37], which identified six areas of program foci: observation skills, empathy, tolerance to uncertainty, cultural sensitivity, team building and collaboration, and wellness and resiliency. However, Alkhaifi et al. studied multiple visual arts-based methods, while our review focused specifically on VTS, and the scope of their review was restricted to undergraduate education. Clinical observation had the strongest evidence of its effectiveness compared to the other competencies. This agrees with our findings, since observational skills were more easily assessed objectively with pre- and post-tests, while the assessment of the other skills was more often based on subjective feedback from students.

### Strengths and Limitations

To our knowledge, this is the first systematic review addressing VTS use in medical education. While we conducted this review using rigorous and established methods, some limitations remain inherent. Although the review of reference lists from included studies and from previous reviews did not reveal a significant body of literature missed by our search strategy, our systematic review remains limited by the keywords and databases used.

Seven of the 11 studies that were included were conducted in USA. This limits the generalization of findings for other geographical contexts, since we may hypothesize that in the USA there is a greater familiarity with VTS methodology and differences in medical curricula.

The marked heterogeneity in the design and reported outcome measures of the studies that were included precluded the meta-analysis of the results.

### Literature gap and further research

Future studies should aim to implement randomized controlled designs, with larger samples, follow-up assessments, and multi-institutional participation. We recommend moving beyond subjective assessments to rigorous pre- and post-course surveys and evaluations, using validated scales to measure outcomes [38]. Additional research is also needed to study VTS in clinical settings and also to examine the best dosage and timing to deliver VTS training in medical education.

## Conclusion

The present study systematically reviewed studies on VTS integration into medical curricula and its effectiveness. The findings consistently indicate that the VTS approach can serve as a vehicle to develop crucial clinical competencies, encouraging more in-depth visual analysis that could be applied when observing a patient. There is a need for further research to deepen our knowledge regarding the role of VTS in medical education, which could include studying VTS in clinical settings, with more robust study designs, longer follow-up, and larger sample sizes.

## Supplementary Information

**Additional file 1.** Search strategies for electronic databases.

**Additional file 2.** Risk of bias assessment- JBI Critical Appraisal Checklists

## Data Availability

The dataset supporting the conclusions of this article is included within the article (and its additional files).

## Abbreviations

VTS: Visual Thinking Strategies
CENTRAL: Cochrane Central Register of Controlled Trials
PRISMA: Preferred Reporting Items for Systematic Reviews and Meta-Analyses
JBI: Joanna Briggs Institute
RCT: Randomized Controlled Trial
USA: United States of America
PGY: Postgraduate year
