# Supplementary material for: Visual Thinking Strategies in medical education: a systematic review
Source: BMC Med Educ. 2023 Jul 27;23:536. doi: 10.1186/s12909-023-04470-3 (PMC10375761; doi:10.1186/s12909-023-04470-3)
Supplement: Supplementary file 1 — Additional file 1. Search strategies for electronic databases. [file 12909_2023_4470_MOESM1_ESM.pdf]

Additional file 1. Search strategies for electronic databases.

a) PubMed Search Strategy

#1	education, medical[MeSH Terms]
#2	schools, medical[MeSH Terms]
#3	academic, medical centers[MeSH Terms]
#4	preceptorship [MeSH Terms]
#5	medical curricul*[Title/Abstract]
#6	students, medical[MeSH Terms]
#7	medical education[Title/Abstract]
#8	medical student[Title/Abstract]
#9	medical school[Title/Abstract]
#10	medicine[Title/Abstract]
#11	medicine[MeSH Terms]
#12	clinical clerkship[MeSH Terms]
#13	education, medical, graduate[MeSH Terms]
#14	education, medical, undergraduate[MeSH Terms]
#15	undergraduate med*[Title/Abstract]
#16	undergraduate stud*[Title/Abstract]
#17	postgraduate med*[Title/Abstract]
#18	postgraduate stud*[Title/Abstract]
#19	#1 OR #2 OR #3 OR #4 OR #5 OR #6 OR #7 OR #8 OR #9 OR<br>#10 OR #11 OR #12 OR #13 OR #14 OR #15 OR #16 OR #17 OR #18
#20	visual thinking [Title/Abstract]
#21	visual skill
#22	visual literacy[Title/Abstract]
#23	visual art*[Title/Abstract]
#24	visual diagnos*[Title/Abstract]
#25	#20 OR #21 OR #22 OR #23 OR #24
#26	#19 AND #25

Filter: 1991-

b) Cochrane Central Register of Controlled Trials (CENTRAL) Search Strategy

#1	MeSH descriptor: [Education, Medical] explode all trees
#2	MeSH descriptor: [Schools, Medical] explode all trees
#3	MeSH descriptor: [Academic Medical Centers] explode all trees
#4	MeSH descriptor: [Preceptorship] explode all trees
#5	(medical curricul*):ti,ab,kw
#6	MeSH descriptor: [Students, Medical] explode all trees
#7	(medical education):ti,ab,kw
#8	(medical student):ti,ab,kw
#9	(medical school):ti,ab,kw
#10	(medicine):ti,ab,kw
#11	MeSH descriptor: [Medicine] explode all trees
#12	MeSH descriptor: [Clinical Clerkship] explode all trees
#13	MeSH descriptor: [Education, Medical, Graduate] explode all trees
#14	MeSH descriptor: [Education, Medical, Undergraduate] explode all trees
#15	(undergraduate med*):ti,ab,kw
#16	(undergraduate stud*):ti,ab,kw
#17	(postgraduate med*):ti,ab,kw
#18	(postgraduate stud*):ti,ab,kw
#19	#1 OR #2 OR #3 OR #4 OR #5 OR #6 OR #7 OR #8 OR #9 OR #10 OR #11 OR #12 OR #13 OR #14 OR #15 OR #16 OR #17 OR #18
#20	visual skill
#21	(visual thinking):ti,ab,kw
#22	(visual literacy):ti,ab,kw
#23	(visual art*):ti,ab,kw
#24	(visual diagnos*):ti,ab,kw
#25	#20 OR #21 OR #22 OR #23 OR #24
#26	#19 AND #25 with Publication Year from 1991 to 2022, in Trials

c) PsycInfo Search Strategy

S1	MA medical education or medical school or medical students or medical curriculum or medical student education or clinical education : Search modes - Boolean/Phrase
S2	MA academic medical center : Search modes - Boolean/Phrase
S3	MA preceptorship or clinical clerkship : Search modes - Boolean/Phrase
S4	MA medicine: Search modes - Boolean/Phrase
S5	AB medical curricul*: Search modes - Boolean/Phrase
S6	AB medical education or medical school or medical students or medical curriculum or medical student education or clinical education: Search modes - Boolean/Phrase
S7	AB medicine: Search modes - Boolean/Phrase
S8	MA graduate medical education: Search modes - Boolean/Phrase
S9	MA undergraduate medical education: Search modes - Boolean/Phrase
S10	AB undergraduate med* or undergraduate stud*: Search modes - Boolean/Phrase
S11	AB postgraduate med* or postgraduate stud*: Search modes - Boolean/Phrase
S12	S1 OR S2 OR S3 OR S4 OR S5 OR S6 OR S7 OR S8 OR S9 OR S10 OR S11: Search modes - Boolean/Phrase
S13	TX visual skill: Search modes - Boolean/Phrase
S14	AB visual thinking: Search modes - Boolean/Phrase
S15	AB visual literacy: Search modes - Boolean/Phrase
S16	AB visual art*: Search modes - Boolean/Phrase
S17	AB visual diagnos*: Search modes - Boolean/Phrase
S18	S13 OR S14 OR S15 OR S16 OR S17 : Search modes - Boolean/Phrase
S19	S12 AND S18: Search modes - Boolean/Phrase
S20	PY 1991-: Search modes - Boolean/Phrase
S21	S19 AND S20: Search modes - Boolean/Phrase
