# Supplementary material for: Visual Thinking Strategies in medical education: a systematic review
Source: BMC Med Educ. 2023 Jul 27;23:536. doi: 10.1186/s12909-023-04470-3 (PMC10375761; doi:10.1186/s12909-023-04470-3)
Supplement: Supplementary file 2 — Additional file 2. Risk of bias assessment- JBI Critical Appraisal Checklists [file 12909_2023_4470_MOESM2_ESM.pdf]

Additional file 2. Risk of bias assessment- JBI Critical Appraisal Checklists

**Checklist for Quasi-Experimental Studies**	Bentwich & Gilbey, 2017 (31)	Klugman et al., 2011(23)	Jasani & Saks, 2013 (24)	Huang et al., 2016 (25)	Agarwal et al., 2020 (26)	Cole et al., 2020[a] (27)	Ho Tiu et al., 2019 (32)
1.Is it clear in the study what is the 'cause' and what is the 'effect' (i.e. there is no confusion about which variable comes first)?	Yes	Yes	Yes	Yes	Yes	-	Yes
2.Were the participants included in any comparation similar?	Yes	Yes	Yes	Yes	No	-	Yes
3.Were the participants included in any comparisons receiving similar treatment/care, other than the exposure or intervention of interest?	Yes	No	Yes	No	No	-	No
4.Was there a control group?	No	No	No	No	Yes	-	No
5.Were there multiple measurements of the outcome both pre and post the intervention/exposure?	No (pre- and post-, single measurement)	No (pre- and post-, single measurement)	No (pre- and post-, single measurement)	No (pre- and post-, single measurement)	No (pre- and post-, single measurement)	-	No
6.Was follow up complete and if not, were differences between groups in terms of their follow up adequately described and analyzed?	No	Unclear	Unclear	No	No	-	Yes
7.Were the outcomes of participants included in any comparisons measured in the same way?	Yes	Yes	Yes	Yes	Yes	-	Yes
8.Were outcomes measured in a reliable way?	Yes	Yes	Yes	Yes	Yes	-	Yes
9.Was appropriate statistical analysis used?	Yes	Yes	Yes	Yes	Yes	-	Yes

[a] only available as a conference abstract

**Checklist for Qualitative Research**	Visscher et al., 2019 (29)	Allison et al., 2017 (30)	Srivastava et al., 2022 (28)
1.Is there congruity between the stated philosophical perspective and the research methodology?	Yes	Unclear	Unclear
2.Is there congruity between the research methodology and the research question or objectives?	Yes	Yes	Yes
3.Is there congruity between the research methodology and the methods used to collect data?	Yes	Yes	Yes
4.Is there congruity between the research methodology and the representation and analysis of data?	Yes	Yes	Yes
5.Is there congruity between the research methodology and the interpretation of results?	Yes	Yes	Yes
6.Is there a statement locating the researcher culturally or theoretically?	No	Yes	No
7.Is the influence of the researcher on the research, and vice- versa, addressed?	No	No	No
8.Are participants, and their voices, adequately represented?	Yes	Yes	Yes
9.Is the research ethical according to current criteria or, for recent studies, and is there evidence of ethical approval by an appropriate body?	Yes	No	Yes
10.Do the conclusions drawn in the research report flow from the analysis, or interpretation, of the data?	Yes	Yes	Yes

**Checklist for Randomized Controlled Trials**	Naghshineh et al., 2008 (22)
1. Was true randomization used for assignment of participants to treatment groups?	No
2. Was allocation to treatment groups concealed?	No
3. Were treatment groups similar at the baseline?	Yes
4. Were participants blind to treatment assignment?	No
5. Were those delivering treatment blind to treatment assignment?	No
6. Were outcomes assessors blind to treatment assignment?	Yes
7. Were treatment groups treated identically other than the intervention of interest?	Yes
8. Was follow up complete and if not, were differences between groups in terms of their follow up adequately described and analyzed?	No
9. Were participants analyzed in the groups to which they were randomized?	Yes
10. Were outcomes measured in the same way for treatment groups?	Yes
11. Were outcomes measured in a reliable way?	Yes
12. Was appropriate statistical analysis used?	Yes
13. Was the trial design appropriate, and any deviations from the standard RCT design (individual randomization, parallel groups) accounted for in the conduct and analysis of the trial?	Yes
